# Associations of Dietary Intake on Biological Markers of Inflammation in Children and Adolescents: A Systematic Review

**DOI:** 10.3390/nu13020356

**Published:** 2021-01-25

**Authors:** Melissa Bujtor, Anne I. Turner, Susan J. Torres, Laura Esteban-Gonzalo, Carmine M. Pariante, Alessandra Borsini

**Affiliations:** 1 Institute for Physical Activity and Nutrition Research, School of Exercise and Nutrition Sciences, Deakin University, Melbourne, VIC 3125, Australia; melissa.bujtor@deakin.edu.au (M.B.); anne.turner@deakin.edu.au (A.I.T.); susan.torres@deakin.edu.au (S.J.T.); 2 Nursing Department, Faculty of Medicine, Autonomous University of Madrid, 28029 Madrid, Spain; laura.esteban@uam.es; 3 Stress, Psychiatry and Immunology Laboratory, Department of Psychological Medicine, Institute of Psychiatry, Psychology & Neuroscience, King’s College, London SE5 9RT, UK; carmine.pariante@kcl.ac.uk

**Keywords:** dietary intake, dietary pattern, macronutrients, biomarkers, inflammation, CRP, cytokine, interleukin, children, adolescent

## Abstract

Background: In children and adolescents, chronic low-grade inflammation has been implicated in the pathogenesis of co- and multi-morbid conditions to mental health disorders. Diet quality is a potential mechanism of action that can exacerbate or ameliorate low-grade inflammation; however, the exact way dietary intake can regulate the immune response in children and adolescents is still to be fully understood. Methods: Studies that measured dietary intake (patterns of diet, indices, food groups, nutrients) and any inflammatory biomarkers in children and adolescents aged 2 to19 years and published until November 2020 were included in this systematic review, and were selected in line with PRISMA guidelines through the following databases: Academic Search Complete, CINAHL, Global Health, Medline COMPLETE and Web of Science–Core Collection. A total of 53 articles were identified. Results: Results show that adequate adherence to healthful dietary patterns such as the Mediterranean diet, or food groups such as vegetables and fruit, or macro/micro nutrients such as fibre or vitamin C and E, are associated with decreased levels of pro-inflammatory biomarkers, mainly c-reactive protein (CRP), interleukin-6 (IL-6) and tumour necrosis factor-alpha (TNF-α), whereas adherence to a Western dietary pattern, as well as intake of food groups such as added sugars, macro-nutrients such as saturated fatty acids or ultra-processed foods, is associated with higher levels of the same pro-inflammatory biomarkers. Conclusions: This is the first systematic review examining dietary intake and biological markers of inflammation in both children and adolescents. A good quality diet, high in vegetable and fruit intake, wholegrains, fibre and healthy fats ameliorates low-grade inflammation, and therefore represents a promising therapeutic approach, as well as an important element for disease prevention in both children and adolescents.

## 1. Introduction

Inflammation is a physiological response to cellular and tissue damage. It is designed to protect the host from bacteria, viruses and infections by eliminating pathogens, promoting cellular repair and restoring homeostatic conditions [1]. However, a prolonged inflammatory state through chronic low-grade inflammation has deleterious effects, including irreparable damage to tissues and organs, and increased risk of disease status [2].

Low-grade inflammation, reflected in the overproduction of acute phase proteins such as C-reactive protein (CRP), pro-inflammatory cytokines such as interleukin-6 (IL-6) and tumour necrosis factor-alpha (TFN-α) has been established as a risk factor for several neuropsychiatric disorders [3], including depression [4,5,6,7] and schizophrenia [8]. Moreover, low-grade inflammation in children and adolescents has been associated with the development of co- and multi-morbid conditions to mental health pathologies [9,10,11,12], including cardiovascular disease [13,14], metabolic syndrome [15], type-II diabetes [16] and obesity [17], therefore making inflammation an important therapeutic target to study, especially for individuals suffering from those conditions.

The potential factors that promote low-grade chronic inflammation are diverse. Stressors such as trauma through adverse childhood experiences, psychosocial stress, as well as modifiable lifestyle sources such as limited physical exercise or smoking are all capable of evoking a deleterious inflammatory response. Increasingly, attention has been given to diet quality as a potential mechanism of action that can exacerbate or ameliorate low-grade inflammation and subsequently influence mental health [18,19]. Certainly, healthy dietary patterns of high quality, such as adherence to a Mediterranean Diet [20], or eating foods such as vegetables and fruit [1], or macro/micronutrients, such as omega-3 poly-unsaturated fatty acids (PUFAs) [21] or vitamins C and E [22], respectively, have been shown to reduce systemic inflammation [23,24]. In observational and interventional studies, a higher quality diet, comprised of these nutrients, has been associated with a reduced risk of adverse mental health in both children [25] and adolescents [26,27]. In contrast, the prevailing Western dietary pattern, which is high in refined grains, red meat, refined sugar and saturated fat, elicits a pro-inflammatory response and increasing levels of circulating inflammatory biomarkers [21].

Moreover, it is well established that a healthy diet in childhood and adolescence is crucial for optimal growth and development and for disease prevention [28]. For example, higher vegetable intake in childhood has been associated with a lower risk of developing mental health pathologies later in life [29], such as depression. In addition, a healthy diet can contribute to the prevention of cardio-metabolic multi-morbidities, often seen in adult patients with neuropsychiatric conditions [30]. As such, modifying dietary intake as early as during childhood and adolescence represents a promising therapeutic strategy in order to maintain a regular immune response, and to reduce the risk of adverse mental health disorders and associated co- and multi-morbid conditions later in life.

Former literature reviews in children and adolescents have focused on various aspects of diet and various biomarkers that, however, are not specifically related to the immune system function and response [31,32,33]. Therefore, to the best of our knowledge this is the first systematic review bringing together the current evidence base from observational and interventional studies investigating associations between dietary intake, by means of dietary patterns, food groups, macronutrients or micronutrients, and biological markers of low-grade inflammation, including CRP, IL-6 and TNF-α among others, in both children and adolescents.

## 2. Materials and Methods

**Figure 1:**
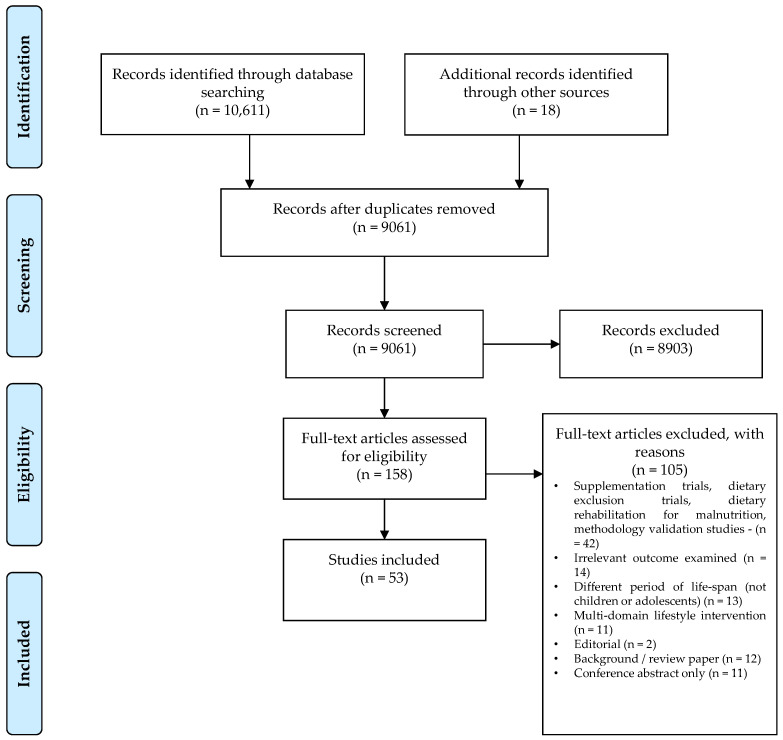
Study selection flow chart including reasons for exclusion of studies during full text screening.

This systematic review complies with the PRISMA Preferred Reporting Items for Systematic Reviews and Meta-analysis guidelines and is registered with PROSPERO (registration code: CRD42020215954) [34]. It comprises papers published until November 2020 and identified across the following databases: Academic Search Complete, CINAHL, Medline COMPLETE, PsycInfo, Embase and Web of Science. Search terms included “dietary intake” OR “dietary pattern” OR “food groups” OR “macronutrients” OR “micronutrients” AND “inflammation” OR “immune biomarkers” OR “inflammatory biomarkers” AND “children” OR “adolescents” and related terms. The selected studies met the following inclusion criteria: peer-reviewed original research articles involving children and adolescents aged 2–19 years, describing associations between inflammation and/or: (a) patterns or indices of dietary intake, such as the Mediterranean Diet, the Western Dietary pattern, and the Healthy Eating Index, (b) food groups such as fruit and vegetables, whole and refined grains and dairy, (c) macronutrients, such as fat and fibre, and (d) micronutrients, such as vitamins A and C. Whereas, exclusion criteria included: non-peer reviewed article publications, studies in a language other than English, animal studies, studies in pregnant women that only examined maternal diet, and supplementation interventions or dietary rehabilitation interventions for malnutrition. The search included studies published until December 2020. Overall, a total of 53 studies met the inclusion criteria for our review (see Figure 1) and were assessed for risk of bias following the Cochrane risk-of-bias (RoB 2.0) for intervention studies, and the Strengthening the Reporting of Observational Studies in Epidemiology (STROBE) checklist for observational studies [35,36], see Supplement S1. The majority of the studies included in this review had a low risk of bias.

## 3. Results

The 53 studies included in this review have been categorized into: (1) dietary patterns and indices (see Table 1), (2) food groups and (3) macronutrients and micronutrients (see Table 2). In particular, 26 studies examined dietary patterns and indices, 15 studies examined food groups, 19 studies examined macronutrients and 7 studies examined micronutrients, while some studies examine more than one category. In total, there were 8 interventional studies, and 45 observational studies, of which 2 were longitudinal [37,38], while the remaining 43 studies were cross-sectional.

### 3.1. Dietary Patterns

Out of the 53 studies, 17 examined a priori dietary patterns which are based on countries dietary guidelines [9]. The examined dietary patterns included the Dietary Approaches to Stop Hypertension (DASH) dietary pattern, low and high glycaemic index dietary patterns, the Mediterranean dietary pattern and the Western dietary pattern. Two studies examined more than one dietary pattern [39,40].

#### 3.1.1. DASH Dietary Pattern

The DASH dietary pattern was examined in one *intervention* study [41] and one *observational* study [40]. The first study found adherence to a DASH diet and lower levels of CRP, in a female cohort of patients with metabolic syndrome [41]. In contrast, the second study did not find an association between adherence to a DASH diet and CRP in a cohort of males and females with type-1 diabetes [40].

#### 3.1.2. Low and High Glycaemic Index Dietary Pattern

Low and high glycaemic index diet patterns were examined in four *interventional* studies [42,43,44,45]. These studies found that adherence to a low glycaemic index diet pattern or to a hypocaloric high glycaemic index diet pattern was associated with lower levels of CRP, both in males and females with obesity.

In particular, the first study found an association between adherence to a low glycaemic index diet and lower levels of CRP and IL-6 in females with obesity or overweight [45]. Another two studies found an association between adherence to both a hypocaloric low and a hypocaloric high glycaemic index diet and lower levels of high sensitivity c-reactive protein (hs-CRP) in males and females with obesity [43,44]. In contrast, a fourth study did not find any associations between low and high glycaemic index diet patterns and inflammatory outcomes in a healthy cohort of male and female participants [42].

#### 3.1.3. Mediterranean Dietary Pattern

The Mediterranean dietary pattern was examined in 10 *observational* studies [39,40,46,47,48,49,50,51,52,53], one of which was also discussed in Section 3.1.1 [40]. These studies found that adherence to a Mediterranean dietary pattern was associated with lower levels of CRP, IL-6, and TNF-α in healthy males and females, whereas the majority of studies conducted in cohorts with underlying pathologies, including type-1 diabetes and obesity, found no associations with inflammatory biomarkers.

Two studies, in particular, found an association between a Mediterranean diet pattern and lower levels of hs-CRP in females only [52], or both in males and females [46], whereas another study found an inverse association between a Mediterranean diet and IL-1, IL2, IL-6 and TNF-α both in males and females [48], all studies were conducted in cohorts of healthy participants. In contrast, one study found a positive association between a Mediterranean diet pattern, and CRP and transforming growth factor beta 1 (TGFβ-1) in males only, but an inverse association with soluble vascular cell adhesion molecule-1 (sVCAM-1) in male and females [47], again the study was conducted in a cohort of healthy participants. Another study found an association between a Mediterranean diet pattern and lower levels of IL-4 and IL-17, but higher levels of IL-33, in both males and females with asthma [49]. In contrast, one study in a type-1 diabetic cohort [40], three studies in obese cohorts [50,51,53], and one study in a healthy cohort [39] did not find any associations.

#### 3.1.4. Western Dietary Pattern (WDP)

The WDP was examined in three *observational* studies [37,39,54]; one study was aforementioned in Section 3.1.3 [39]. These studies found that adherence to a WDP was associated with higher levels of CRP in a cohort of healthy males and females.

The first study was longitudinal and found an association between WDP adherence at 14-years and higher levels of hs-CRP at 17-years both in healthy males and females [37]. The remaining two studies found an association between WDP adherence, and higher levels of CRP and IL-6 in this case in female cohorts of healthy participants [39,54].

### 3.2. Indices

Out of the 53 studies, 11 examined dietary indices, including the dietary inflammatory index, healthy eating index and the diet quality index. One study examined both a pattern and an index [51], while another study examined two patterns and one index [40].

#### 3.2.1. Dietary Inflammatory Index (DII)

The DII, a tool that assesses the inflammatory potential of a diet [32], was examined in 6 *observational* studies [38,51,55,56,57,58], one of which was also discussed in Section 3.1.3 [51]. These studies found that adherence to a pro-inflammatory diet (indicated by a higher score in the DII) was associated with higher levels of hs-CRP, IL-6 and TNF-α in healthy males and females.

In particular, four studies found a positive association between adherence to a pro-inflammatory diet (indicated by a higher score in the DII) and IL-6 [55,57], IL-1, IL-2, interferon gammon and sVCAM-1 in healthy males and females [58], but also a positive association between DII and CRP in males and females with obesity [51]. However, two studies did not find any associations between the DII and CRP [38], or IL-6, MCP-1 or TNF-α [56] in neither healthy males or females.

#### 3.2.2. Healthy Eating Index (HEI)

The HEI, a tool that assesses adherence to the Dietary Guidelines of Americans in any given year, was examined in three *observational* studies [40,59,60]. One of which was also discussed in Section 3.1.1 and Section 3.1.3 [40]. The first study found that a higher score in the HEI (healthy diet) was associated with lower levels of CRP in females, but not males from a healthy cohort [59]. In contrast, two studies found that moderate HEI scores (moderately healthy diet) were not associated with CRP or IL-6 in males or females in cohorts of patients with type-1 diabetes [40,60].

#### 3.2.3. Diet Quality Index (DQI)

The DQI, a composite individual-level diet quality indicator that enables cross-cultural diet quality comparisons was examined in two *observational* studies [61,62]. Neither of the studies found an association between the DQI and CRP [61,62] or IL-1, IL-6, IFN-y and TNF-α [62]. This suggests a lack of sensitivity associated with the DQI as a measure of inflammatory status.

### 3.3. Food Groups

Out of the 53 studies, 15 examined food groups, in particular: (1) vegetables and fruits, (2) dairy, (3) meat, seafood and eggs, (4) whole and refined grains, and (5) added sugars.

#### 3.3.1. Vegetables and Fruits

Vegetable and/or fruit intake was examined in nine observational studies [47,59,60,63,64,65,66,67,68], two of which were also discussed in Section 3.2.2 [59,60]. These studies found that high dietary intake of vegetables and/or fruits was associated with lower levels of CRP and IL-6 (both in healthy males and females), TNF-α (only in healthy females), and IL-17F (both male and female patients with asthma).

In particular, three studies found an association between vegetable and fruit intake and lower levels of CRP [63,64,65], and IL-6 and TNF-α [66], both in healthy males and females, as well as in males and females with obesity [69]. In contrast, one study found an association between vegetable and fruit intake and lower levels of hs-CRP in healthy females, but not in males [59]. While another found an association between vegetable and fruit intake and higher levels of CRP in males, but not in females, lower levels of IL-4 in males and females, and lower levels of TNF-α in females, but not in males, and higher levels of IL-10 in males and females, in a cohort of healthy participants [47]. While one study found an association between fruit and vegetable intake and lower levels of IL-17F in males and females from a cohort of patients with asthma [67], another study found no associations between vegetable and fruit intake and CRP in a cohort of male and female patients with type-1 diabetes [60].

#### 3.3.2. Dairy

Dairy was examined in seven observational studies [47,60,63,64,67,68,69], six of which were also discussed in Section 3.3.1 [47,60,63,64,67,69], and one in Section 3.1.3 [47]. One study found an association between dairy intake and higher levels of IL-17F in males and females, from a cohort of patients with asthma. However, the majority of studies did not find an association between dairy intake and CRP or IL-6 in males or females from healthy cohorts.

One study found an association between dairy intake and higher levels of IL-17F in males and females from a cohort of patients with asthma [67] while a second study found an association between dairy intake and higher levels of IL-6, Il-10 and TGFβ-1 in females, but not in males, and IL-1 in both males and females, and IL-5 in males but not in females in a healthy cohort [47]. The remaining five studies did not find any associations between dairy intake and CRP and IL-6 in males and females from healthy cohorts [60,63,64,68,69].

#### 3.3.3. Meat, Seafood and Eggs

Meat, seafood and eggs were examined in seven observational studies [47,60,63,64,67,69,70], six of which were also discussed in Section 3.3.1 and Section 3.3.2 [47,60,63,64,67,69], and one in Section 3.1.3 [47]. The majority of studies did not find an association between dietary intake of meat, seafood and eggs and CRP, IL-6 and TNF-α in males and females from healthy cohorts.

Two studies, in healthy populations found an association between meat intake and higher levels of IL-2, IL-10 [47] and IL-6 [70] in healthy males and females, however the remaining five studies did not find any associations between meat, seafood or eggs intake and CRP, IL-6 and TNF-α [60,63,64,67,69].

#### 3.3.4. Whole and Refined Grains

Whole and refined grains were examined in one intervention [71] and six observational [47,63,64,67,69,72] studies, six of which were also discussed in Section 3.3.1, Section 3.3.2 and Section 3.3.3 [47,63,64,67,69], and one in Section 3.1.3 [47]. These studies found that wholegrain dietary intake was associated with lower levels of CRP and IL-17F in males and females from cohorts with and without underlying pathologies. In contrast, results on the association between refined grain intake and inflammatory markers remain inconclusive.

In particular, the first study found an association between wholegrain intake and lower levels of CRP in a female only cohort of participants with obesity [71]. The second study found an association between wholegrain intake and lower levels of IL-17F in males and females from a cohort of patients with asthma [67]. A third study found an association between wholegrain intake and lower levels of CRP, in males and females from a healthy cohort [72].

The remaining studies examined refined grains in healthy populations. One study reported an association between refined grain intake and lower levels of hs-CRP in males and females [64], while another study found an association with higher levels of CRP, only in females but not in males [63]. A third study found refined grains were associated with lower levels of IL-6 and IL-10 in males and females [47]. Lastly, one study found no association between refined grain intake and CRP in males and females [69].

#### 3.3.5. Added Sugar and Sugar Sweetened Beverages (SSB)

Added sugars (snacks—candy, jams, spreads, sugar sweetened beverages, fruit juice) were examined in one interventional [73] and eight observational [50,60,63,65,66,67,69,74] studies, two of which were also discussed in Section 3.2.1, Section 3.2.2 and Section 3.2.3 [67,69]. The majority of studies did not find an association between added sugar intake and CRP, for males or females from cohorts with healthy participants, as well as for patients with inflammatory bowel disease, Type-1 diabetes and obesity.

In particular, the first study found glucose intake decreased levels of hs-CRP, compared with fructose in a cohort of male and female patients with non-alcohol fatty liver disease [73]. Another study found an association between higher SSB intake and higher levels of CRP in males and females from a cohort of healthy participants [74]. Another two studies found an association between SSB intake and higher levels of CRP in females, but not in males in a cohort of healthy participants [63], and IL-17F in both males and females from a cohort of patients with asthma [67]. Five studies found no association between added sugar intake and CRP, IL-6 and TNF-α from cohorts of healthy participants [66,69], patients with inflammatory bowel disease, Type-1 diabetes [60] and obesity [50].

### 3.4. Macronutrients

A total of 19 studies examined macronutrients, including (1) fats and (2) fibre.

#### 3.4.1. Fats

Fat was examined in 13 observational studies [39,47,50,59,60,64,70,75,76,77,78,79,80], 8 of which were also discussed in Section 3.1.3 [39,50], Section 3.1.4 [39], Section 3.2.2 [59] Section 3.3.1 [59,60,64], Section 3.3.2 [60,64], Section 3.3.3 [60,64,70], Section 3.3.4 [60,64], and Section 3.3.5 [50,60]. The studies found that saturated fatty acids (SFAs) intake was associated with higher levels of CRP, IL-6, Transforming growth factor-beta and sVCAM-1, while monounsaturated fatty acid (MUFA) intake was associated with lower levels of IL-6, and omega-3 PUFA intake was associated with lower levels of plasminogen activator inhibitor-1, in males and females from cohorts of healthy participants.

These studies found an association between SFAs and higher levels of CRP in females, but not in males from a cohort of healthy participants [59], as well as in male and female obese participants [70]. In contrast, one study found a positive association between SFA intake and CRP in males, but not in females from a cohort of healthy participants [67]. Another study found an inverse association between dietary intake of MUFA and IL-6, as well as between omega-3 PUFAs and plasminogen activator inhibitor-1, in a female cohort of healthy participants [39]. Another study found a positive association between MUFA:SFA ratio and IL-6 in males and females, TGFβ-1 in females but not in males, and sVCAM-1 in males but not in females, from a cohort of healthy participants [47]. The same study also found an inverse association between SFA and sVCAM-1 in females but not in males [47]. Lastly, seven studies found no associations between SFA and CRP [60,76] and total fat and CRP [50,64,75,79,80] or IL-6 [80].

#### 3.4.2. Fibre

Fibre was examined in one *intervention* [81] and nine *observational* [50,59,64,76,78,82,83,84,85] studies, five of which were also discussed in Section 3.1.3 [50], Section 3.2.2 [59], Section 3.3.1 [59,64], Section 3.3.2 [64], Section 3.3.3 [64], Section 3.3.4 [64], Section 3.3.5 [50], and Section 3.4.1 [50,76]. Results show that, while six studies did not find an association between fibre intake and CRP in males or females from healthy cohorts, four studies found an association between fibre intake and lower levels of CRP, TNF-α, plasminogen activator inhibitor-1, and monocyte chemoattractant protein-1 in males and females, from cohorts of healthy participants.

The first study found an association between dietary fibre intake and higher levels of TNF-α in males and females from a cohort of healthy participants [81]. Another two studies in healthy participants found an association between dietary fibre intake and lower levels of CRP in females, but not in males [59], or both in males and females [85]. A fourth study found that total fibre intake was inversely associated with plasminogen activator inhibitor-1, while insoluble fibre was inversely associated with plasminogen activator inhibitor-1 and monocyte chemoattractant protein-1, in males and females from a cohort of overweight participants [86]. Lastly, six studies found no associations between fibre intake and CRP in males or females from cohorts of healthy participants [64,78,82,83,84], or in obese participants [50].

### 3.5. Micronutrients

A total of seven observational studies examined various micronutrients [39,59,66,79,87,88,89]. Three of the studies were also discussed in Section 3.2.2 [59], Section 3.3.1 [59,66] and Section 3.4.1 [59,79]. These studies found an association between dietary intakes of vitamins and lower levels of CRP and IL-6, while sodium was associated with higher levels of TNF-α, in males and females from a cohort of healthy participants [87].

Vitamin C intake was associated with lower levels of hs-CRP and IL-6 in males and females from a cohort of healthy participants. [66]. Dietary intake of beta-carotene was associated with lower levels of IL-6 and TNF-α [66], while vitamins A and E were inversely associated with hs-CRP, in males and females from cohorts of healthy participants [59]. Magnesium was inversely associated with hs-CRP in healthy male and female participants [88] and positively associated with IL-6 in healthy females [39]. One study examined sodium intake and found a positive association with TNF-α in healthy males and females [87]. However, two studies found no associations between micronutrient intake and CRP in males and females from healthy populations [79,89].

## 4. Discussion

**Figure 2:**
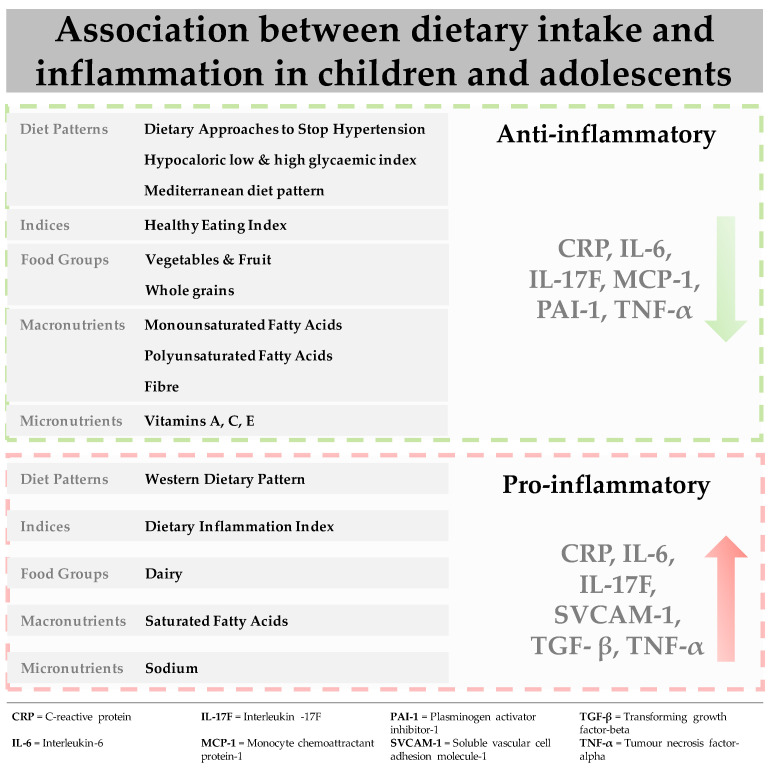
Association between dietary intake and biomarkers of inflammation in children and adolescents.

This review provides the first evidence for the association between dietary intake (dietary patterns, food groups, macronutrients and micronutrients) and biological markers of inflammation in children and adolescents. The main results (Table 1 and Table 2, Figure 2) indicate that adequate adherence to healthful dietary patterns, such as the DASH diet, low glycaemic index diets and the Mediterranean diet are associated with decreased levels of biomarkers, including CRP, IL-6 and TNF-α. Among the individual constituents of these diets, vegetable and fruit intake and wholegrains, as well as healthy fats were associated with a favourable inflammatory response. In contrast, a Western dietary pattern, as well as its separate constituents including saturated fatty acid, elicited a pro-inflammatory response increasing levels of pro-inflammatory biomarkers, such as CRP, IL-6, TNF-α and sVCAM-1. Associations across the studies included in this review were attenuated by gender, as well as the presence of underlying pathologies, independent of dietary intake.

Traditionally diet–disease relationships have been examined by focusing on nutrients or food groups, which can be limiting. Foods are typically eaten in combination, and nutrients have both synergistic and antagonistic biochemical interactions [90]. More recently, dietary patterns that capture the whole diet, involving the combination of foods and nutrients have been examined [91]. The most examined dietary pattern in this review was the Mediterranean diet and in observational studies conducted in healthy populations adequate to high adherence resulted in decreased levels of pro-inflammatory biomarkers [46,48,52]. Similar results were found for studies examining low glycaemic index diets in obese populations [43,44,45] and one intervention study in females with metabolic syndrome which examined the DASH diet [41].

The mechanisms by which these healthful dietary patterns affect the inflammatory process are largely underexplored [18]. It has been hypothesised that the protective effect of these patterns may be derived from the anti-inflammatory properties of their constituents [92]. The Mediterranean diet is characterized by high intakes of vegetables, fruit, wholegrains, legumes, nuts, fish and low-fat dairy, and low intakes of red meat and adequate intakes of healthy fats [93]. As such, the diet is rich in antioxidants, folate, and flavonoids which are anti-inflammatory. The high dietary fibre content supports gut health and the growth of microbial species which potentially regulate the inhibition or production of pro-inflammatory chemokines and cytokines [94]. Omega-3 PUFAs, found in high concentration in oily fish such as salmon, have been shown to regulate the immune response by inhibiting the activation of pro-inflammatory pathways and reducing cytokine expression [3]. High-dose eicosapentaenoic acid has been shown to improve cognitive symptoms in Attention deficit hyperactivity disorder (ADHD) youth with low baseline levels [95,96], while research in animal models has demonstrated inflammation-induced reductions in neurogenesis can be prevented through omega-3 PUFAs intake [97]. Lastly, sodium intake has been implicated in the regulation of the immune response [98]. The DASH diet, which is similar to the Mediterranean diet, with a greater focus on minimal red meat and processed foods, also restricts sodium intake [99]. Similar to sodium, the studies included in this review also examined other dietary components with strong anti-inflammatory properties. For example, studies showed that high intakes of vegetables and fruit [59,63,64,65,66,67,69], and whole-grains [67,72] resulted in lower levels of inflammatory biomarkers, such as CRP, IL-6 and TNF-α. The same was for various micronutrients such as beta-carotene [66] and vitamins C and E [39] all of which are considered to have anti-inflammatory properties [100].

In contrast to healthful dietary patterns, studies that examined the Western dietary pattern, characterised by high amounts of refined grains, red meat, high fat dairy, ultra-processed food intake and trans fatty acids while being low in omega-3 PUFAs [101,102] showed positive associations with pro-inflammatory markers [37,54]. Similarly, in the studies that examined the DII, diets with high inflammatory potential, inducing a higher inflammatory response were positively associated with pro-inflammatory biomarkers in males and females [51,55,57,58]. In terms of separate components comprising these diets, saturated fatty acid intake is known to be a significant pro-inflammatory contributor that stimulates IL-6 secretion, while in contrast high intakes of healthier fats, such as omega-3 PUFAs found in oily fish can inhibit the inflammatory response [103]. Across the studies that examined saturated fatty acids in this review there was evidence for a positive association between saturated fatty acid and CRP, as well as various cytokines, IL-6, sVCAM and sICAM [59].

As demonstrated in this review, the relationship between diet and inflammation is attenuated by a number of different factors [1,104,105]. For example, gender differences were evident across the studies included in this review, however, they were not specific to one particular diet, food group, macro- or micronutrient. Nor where they specific to any inflammatory biomarker. We hypothesize the gender difference is potentially attributable to the influence of hormones. Sex hormones affect immune function, whereby estrogens stimulate auto-immunity and androgens exhibit protective properties [106,107,108,109]. Other evidence indicates genetic, epigenetic and environmental factors may also contribute to gender differences; however, no included studies explored these potential associations [109,110]. Furthermore, we acknowledge the heterogeneity in the cohorts of the studies included in this review, and as such some associations could have been confounded by the sample population. A number of studies examined cohorts with underlying pathologies, with overweight/obesity [42,43,44,45,50,51,53,70,71,75,81,86], and type-1 diabetes [40,60,82] being the most studied. In overweight and obese populations excess adipose tissue has been linked to an increase in sub-chronic levels of key pro-inflammatory cytokines, mainly CRP, IL-6 and TNF-α [111], and this may prevent or attenuate any potential therapeutic effect exerted by a healthful diet [112]. Taken together, physiologic mechanisms specific to disease state or population may inhibit any beneficial influence of a healthy dietary pattern, potentially explaining the lack of significant associations in the studies discussed in our review [82].

With regards to methodology, it is widely accepted that nutrition epidemiology studies are affected by reporting bias. Imprecision in the measurement of dietary intake is often observed, particularly in children and adolescents, which can cause the over- or under- estimation of the impact of exposure [113,114,115]. Moreover, the measurement tools themselves are often flawed, for example the KidMed questionnaire, used to evaluate the level of adherence to the Mediterranean diet, has a strong bias toward healthy foods and as such may not adequately capture hidden constituents such as sodium [116]. These methods also do not consider the biological effect of food (intake versus absorption) [117]. More recently, several studies have examined associations between biological markers of dietary intake and pro-inflammatory biomarkers. Using these methods, an inverse association between fatty acid composition in erythrocytes and pro-inflammatory biomarkers (IL-1β and IL-6) has been observed in children and adolescents [118], and higher omega-6/omega-3 PUFAs ratio has been associated with higher levels of inflammation [119] and subsequent adverse mental health effects [120]. These biological, rather than self-reported, dietary measures are a more accurate and reliable way of investigating dietary intake, which should be more often used in future research studies [121].

Lastly, there is currently no consensus regarding the inflammatory biomarkers best used to represent chronic low-grade inflammation in children and adolescents, and biomarker measurement error such as sampling, storage and laboratory errors also cannot be excluded [122]. The majority of the studies in this review used a single static measurement of inflammation, however, inflammatory markers owing to their role in homeostasis and immune response are by nature not static and when measured in the fasting state are recognised as being insensitive, and producing highly variable results [2]. Multiple, non-fasting state measures would provide for more accurate and meaningful outcomes.

## 5. Conclusions

In conclusion, our review shows that healthful dietary patterns such as the Mediterranean diet, high in vegetables, fruit, wholegrains, legumes, nuts, fish and low-fat dairy, alongside moderate to low consumption of meat and healthy fats, have an inverse association with pro-inflammatory biomarkers, particularly CRP, IL-6 and TNF-α, in children and adolescents. Components of healthful diets that were examined separately, including food groups such as vegetables and fruit, or macronutrients such as fibre, or micronutrients such as Vitamins A, C and E also have inverse associations with pro-inflammatory biomarkers. In contrast, the Western dietary pattern, as well as its individual components including macronutrients such as saturated fatty acids, micronutrients such as sodium and ultra-processed foods, increases levels of the same pro-inflammatory biomarkers. Further interventional research is needed to establish the strength of associations between dietary intake and inflammatory biomarkers, and ultimately to develop a better understanding of the biological mechanisms underlying such associations.

## Figures and Tables

**Table 1:** Summary of studies investigating the associations between dietary patterns/scores/indices and biological markers of inflammation in children and adolescents.

Author and Country (* also in Table 2)	Sample Characteristics	Pathology (Present or Outcome)	Dietary Pattern or Index (Intervention Period if Applicable)	Components of Dietary Pattern or Indices	Biological Markers of Inflammation	Main Findings ↑ Increase/Positive Association ↓ Decrease/Negative or Inverse Association ↔ No Association
Experimental						
Damsgaard et al. (2012) (Europe) [42]	*n* = 253 13.2 (10.6–16.2) female 12.8 (10.3–15.2) male 51% female	Parents were overweight/obese Present	Randomized to 1 of 5 diet patterns: 1. High Protein Diet Pattern 2. Low Protein Diet Pattern 3. HGI Diet Pattern 4. LGI Diet Pattern 5. Control (6 months)	High protein/low protein High GI/low GI	hs-CRP	Patterns—high protein, low protein, high GI, low GI, control: ↔ No associations
Iannuzzi et al. (2009) (Italy) [43]	*n* = 26 7–13 years 54% female	Obese Present	Hypocaloric HGI Diet Pattern Hypocaloric LGI Diet Pattern	HGI: Energy intake 30% < required for weight maintenance, 25–30% fat, 15–20% protein, 50–60% carbohydrate, high glycaemic index. LGI: same as HGI diet but mean daily estimated index was 60	CRP	HGI intake: CRP ↓ LGI intake: CRP ↓
Parillo et al. (2012) (Italy) [44]	*n* = 22 8–13 years 54% female	Obese Present	Hypocaloric HGI Diet Pattern Hypocaloric LGI Diet Pattern	HGI: Energy intake < 30% less than reqd. for weight maintenance, 25–30% fat, 15–20% protein, 50–60% carbohydrate, fibre intake 0.5 g/kg and high glycaemic index of 90. LGI: Same as for HGI diet, but mean daily estimated glycaemic index was 60.	hs-CRP	HGI intake: CRP ↓ LGI intake: CRP ↓
Rouhani et al. (2016) (Iran) [45]	*n* = 50 Exp-25, 13.28 ± 0.16 Cont-25, 13.93 ± 0.24 100% female	Overweight/obese Present	LGI Diet Pattern HND (10 weeks)	LGI: carbohydrate containing foods from a list of LGI grains, fruits, vegetables, dairy and high glycaemic foods to be avoided (moderately calorie restricted) HND: based on healthy eating guidelines	CRP, Il-6	LGI intake: IL-6 and CRP ↓, HND intake: Il-6 ↓ All other ↔
Saneei et al. (2014) (Iran) [41]	*n* = 60 (30 exp. 30 cont.) 14.2 ± 0.7 100% female	Metabolic syndrome Present	Randomized to: DASH Dietary Pattern Typical Iranian Diet (cross-over trial: 6-wk cycle, 4 wk wash-out)	DASH Diet Typical Iranian Diet	CRP, IL-2, IL-6, TNF-α,	DASH, compared to typical Iranian diet: CRP ↓ All other ↔
Observational						
Agostinis-Sobrinho et al. (2018) (Columbia and Portugal) [46]	*n* = 1462 13.5 ± 2.1 43% female	NA	MDP	Kidmed (16 questions, maximum score 12): Group 1—optimal adherence (≥8, optimal MedDiet); and Group 2 and 3—Low adherence (4–7, improvement needed (adjust intake to Med) or ≤3, very low diet quality)	hs-CRP	Optimal adherence to MDP + High MF/High CRF: hs-CRP ↓ Optimal adherence to MDP + Low MF/Low CRF: hs-CRP ↑ Low adherence to MedDiet + Low MF/Low CRF: hs-CRP ↑
Almeida-de-Souza et al. (2015) (Portugal) [55]	*n* = 329 15.00 (13.0–16.0) 56% female	NA	DII	DII score was calculated considering 31 food parameters, expressed as tertiles: Tertile 1—low (<−1.34) Tertile 2—medium (−1.34 to 1.41) Tertile 3—high (>1.41)	CRP, IL-6	DII: 0.57 ± 0.92–2.07 3rd Tertile compared to 1st: Il-6 ↑ CRP ↔
Arouca et al. (2017) * (Europe) [47]	*n* = 464 14.79 ± 1.24 53% female	NA	MDP	MDP: MD score (0–9 points, higher scores indicating greater adherence) Positive components: (1) high ratio of monounsaturated to saturated dietary lipids (mainly olive oil), (2) vegetables, (3) fruits and nuts, (4) fish (5) cereals (6) of pulses Negative components: (7) meat and meat products (8) milk and dairy products, and (9) any consumption of alcohol (deducted in this study)	CRP, IL-1, IL-2, IL-4, IL-5, IL-6, Il-10, sVCAM-1, sICAM-1, sE-selectin, TNF-α, TGFβ-1	MDS (mean 4.23 ± 1.49 boys, 4.19 ± 1.43 girls): CRP, TGFβ-1 ↑ (boys), sVCAM-1 ↓ (all) All other ↔
Carvalho et al. (2018) (Multi-country) [48]	*n* = 242 14.4 ± 1.1 56.61% female	NA	MDP	MDP: modified MD score (0–8 points, higher scores indicating greater adherence) Positive components: (1) high ratio of monounsaturated to saturated dietary lipids (mainly olive oil), (2) vegetables, (3) fruits and nuts, (4) fish (5) cereals (6) of pulses Negative components: (7) meat and meat products (8) milk and dairy products	CRP, IL-1, IL-2, IL-4, IL-6, TNF-α,	MDS: 4.2 ± 1.5 High adherence vs. low adherence: IL-1, IL-2, IL-6, TNF-α ↓ (unadjusted)
Chan et al. (2015) (Australia) [61]	*n* = 1419 14 years, 49% female *n* = 843 17 years 53.5% female	NA	DGI-CA	Modified DGI-CA: score 0–100 points, scores closer to 100 represent maximum compliance, item 10. alcohol excluded Eleven indicators (1) wide variety of foods, (2) serves of vegetables, legumes, fruit (3) serves of breads and cereals (4) lean meat/fish/poultry (5) milk/yoghurt/cheese (6) plenty of water (7) limited SFA/moderate total fat (8) low salt foods (9) prevent weight gain (10)	hs-CRP	DGI-CA 14-yrs 47.1 ± 10.2; and DGI-CA17-yrs 47.7 ± 11.0: ↔ No associations reported
Coheley et al. (2019) (USA) [56]	*n* = 323 11.38 ± 1.23 57% female	NA	DII	DII calculated from 27 food parameters available (of 45 recognised in the DII score) from the 3 day food diary, expressed as levels of inflammatory potential: [<−1.34]—low [−1.34 to 1.41]—medium [>1.41]—and high	IL-6, MCP-1, TNF-α	DII: score 0.59 ± 1.36 (pro-inflammatory): ↔ No associations reported
del Mar Bibiloni (2013) * (Spain) [39]	*n* = 219 14.9 ± 1.3 100% female	NA	WDP MDP	WDP—yoghurt and cheese, dairy desserts, red meats, poultry, sausages, eggs, bread, cereals, pasta, rice dishes, pizza, fruit juices, canned fruits, nuts, soft drinks, high-fat foods, other oils and fats, sweets and chocolates MDP—yoghurt and cheese, red meat, poultry, fish and seafood, eggs, legumes, pasta, fresh fruit, fruit juices, vegetables, potatoes, tubercles and olive oil Participants were categorised across tertiles of the two patterns	hs-CRP, IL-6, PAI-1, TNF-α	WDP score: Il-6 ↑, ↔ All other MDP score: ↔ No associations reported
Douros et al. (2018) (Greece) [49]	*n* = 70 Exp-44, 8.9 ± 2.4, 41% female Cont-26, 8.6 ± 2.1, 35% female	Asthma Present	MDP	Kidmed (16 questions, maximum score 12): Optimal adherence: ≥8, optimal MedDiet Average adherence: 4–7 Low adherence: ≤3	IL-4, IL-17, IL-33	KidMed (mean 5.70 ± 1.94): Asthmatic only: IL-4, Il-17 ↓ Il-33 ↑ Control: ↔ No associations reported
Karampola et al. (2019) * (Greece) [50]	*n* = 142: Exp-71 (28 overweight/43 obese) Cont-71 13.4 ± 1.46 46% female	Overweight/obese Present	MDP	KidMed (16 questions, maximum score 12): Group 1—optimal adherence (≥8, optimal MedDiet); Group 2 and 3—Low adherence (4–7, improvement needed (adjust intake to Med) or ≤3, very low diet quality)	hs-CRP	KidMed—obese—5, overweight—7, controls—6: ↔ No associations reported
Khayyatzadeh et al. (2018) (Iran) [54]	*n* = 670 14.5 ± 1.5 100% female	NA	WDP	WDP: high in refined grains, snacks, red meats, poultry, fish, organ meat, pizza, fruit juices, industrial juice and compote, mayonnaise, nuts, sugars, soft drinks, sweets and desserts, coffee and pickle	hs-CRP	WDP (higher adherence): hs-CRP ↑
Kurklu et al. (2019) (Turkey) [57]	*n* = 343 10–16 years 63% female	Metabolic syndrome Outcome	DII	DII calculated from 31 food parameters available (of 45 recognised in the DII score) from the 3 day food diary, expressed as levels of inflammatory potential: Quartile 1 = 1.04–3.19 Quartile 2 = 3.19–3.80 Quartile 3 = 3.81–4.31 Quartile 4 = 4.32–5.11	CRP, IL-6, TNF-α	DII (range: 1.04–5.11, average 3.6 ± 0.82): 4th quartile compared to 1st quartile: Il-6 ↑ ↔ All other
Lazarou et al. (2010) (Greece) [51]	*n* = 83 9.2 ± 1.7 50% female	Obesity Present	MDP DII	KidMed (16 questions, maximum score 12): 0–3: Poor quality 4–12: at least average quality Inflammatory Foods Index: composed of nine foods/food groups	hs-CRP	KidMeD: CRP < 0.10 mg/dL: 33% poor quality, 67% at least average quality CRP > 0.10 mg/dL: 31% poor quality, 69% at least average quality MDP: ↔ No associations reported DII (high score): hs-CRP ↑
Liese et al. (2017) (USA) [40]	*n* = 2520 14.2 ± 3.0 50% female	Type-1 Diabetes Present	DASH dietary pattern HEI-2010 MDP-modified KidMed TAC	Dietary indices were used to evaluate diet quality in this study; DASH, HEI2010, KidMed and TAC All indices were coded based on food item, food group and nutrient data	CRP, IL-6	DASH (43; possible range 0–80): ↔ No associations reported HEI-2010 (55; possible range 0–100): ↔ No associations reported Modified KidMed (3.7; possible range 3–12): ↔ No associations reported Total Antioxidant Capacity (TAC): ↔ No associations reported
Navarro (2017) * (Spain) [59]	*n* = 571 6.8 years 53% female	NA	HEI	HEI Pattern 1: fibre, fruit, fruit and vegetable, and vitamin A and E intakes Pattern 2: fat intake, particularly monounsaturated and polyunsaturated intakes	hs-CRP	HEI (higher score 62.8 ± 10.3 females): hs-CRP ↓ (females only, 3rd tertile hs-CRP) Pattern 1: hs-CRP ↓ (female only) Pattern 2: ↔ No associations reported
Oddy et al. (2018) (Australia) [37]	*n* = 843 14 years 51% female 3-year follow-up	Depression Outcome	WDP (compared to HDP)	WDP: take-away and processed foods, red and processed meats, full-fat dairy, fried potatoes, refined grains, confectionary, soft drink, crisps, sauces and dressings HDP: wholegrains, fruit, vegetables, legumes and fish	hs-CRP	WDP adherence (14-yrs): hs-CRP (17-years) ↑ HDP adherence (14-yrs): hs-CRP (17-yrs) ↓
Sanjeevi et al. (2018) * (USA) [60]	*n* = 136 12.7 ± 2.6 % female not reported	Type-1 Diabetes Present	HEI: representing adherence to Dietary Guidelines of Americans 2015 (diet quality) (Secondary analysis of a trial that aimed to increase intake of whole plant foods in experimental group—18-month behavioural intervention trial)	Index: HEI-2015 comprised of twelve component scores summed to obtain total score 0–100. Higher score indicates higher adherence to DGA 2015	CRP	HEI (46.05 ± 11.70): ↔ No associations reported
Sen et al. (2017) (USA) [38]	*n* = 922 3.1 years and 7.7 years % female not reported	NA	DII	DII derived from FFQ and expressed as quartiles, low to high.	hs-CRP	DII (highest quartiles—early childhood): hs-CRP, (mid childhood) ↔ No associations reported
Shivappa et al. (2016) (Europe) [58]	*n* = 532 12.5–17.5 55% female	NA	DII	DII derived from 25 food parameters (of 45 recognised in the DII score) and expressed levels of inflammatory potential: Tertile 1—low inflammation Tertile 2—medium inflammation Tertile 3—high inflammation	CRP, IL-1, IL-2, IL-6, IL-4, IL-10, IFN-y, sICAM, sVCAM, TNF-α	DII (Tertile 3): IL-1, IL-2, IFN-y, TNF-α, sVCAM ↑ ↔ All other
Sureda et al. (2018) (Spain) [52]	*n* = 364 12–17 years 60% female	NA	MDP	MDP: MD score (0–9 points, higher scores indicating greater adherence) Energy-adjusted, daily consumption values of: legumes, cereals and roots (including bread and potatoes), fruit (including nuts), vegetables, fish, meat (and meat products) and milk (and milk products). Alcohol considered null. Summed then converted to the relative percentage of adherence: Above median value (≥50%) Under median value (<50%)	hs-CRP, PAI-1, TNF-α	Females—41% above median (average adherence): Med Diet Score: hs-CRP ↓, ↔ All other Males—46% above median (average adherence): Med Diet Score: ↔ No associations reported
Vyncke et al. (2013) (Europe) [62]	*n* = 1804 (biomarker sub-sample 552) 14.7 ± 1.2 53% female	NA	DQI-A	DQI-A score 0–100 points, scores closer to 100 represent maximum compliance derived from intake of: (1) water, (2) bread and cereals, (3) grains and potatoes, (4) vegetables, (5) fruit, (6) milk products, (7) cheese, (8) meat, fish, eggs, and substitutes, (9) fats and oils	hs-CRP, IL-1, IL-6, IFN-y, TNF-α	DQI-A (range 11.1 to 82.5, median 55.0): ↔ No associations reported
Yilmaz et al. (2019) (Turkey) [53]	*n* = 95 10–18 years 56% female	Obesity (Present) Cardiovascular Risk Factors (Outcome)	MDP	KidMed (16 questions, maximum score 12): Optimal adherence: ≥8, optimal MedDiet Average adherence: 4–7 Low adherence: ≤3	hs-CRP	Med Diet Quality (low adherence—60% of participants): ↔ No associations reported

CRP—C-reactive protein; DASH—Dietary Approaches to Stop Hypertension; DGA—Dietary Guidelines of Americans; DGI-CA—Dietary Guidelines Index for Children and Adolescents; DII—Dietary Inflammatory Index; DQI—Diet Quality Index; DQI-A—Diet Quality Index-Adolescents; F&V—Fruit and Vegetable; FFQ—Food Frequency Questionnaire; GI—Glycaemic Index; HDP—Healthy Diet Plan; HEI2010—Healthy Eating Index 2010; HGI—High Glycaemic Index; HND—Healthy Nutritional Diet; hs-CRP—high-sensitivity C-reactive protein; IL-1—Interleukin-1; IL-10—Interleukin-10; IL-2—Interleukin-2; IL-4—Interleukin-4; IL-5—Interleukin-5; IL-6—Interleukin-6; Kidmed—Mediterranean Diet Quality Index for children and teenagers; LGI—Low Glycaemic Index; MCP-1—Monocyte Chemoattractant Protein-1; MDP—Modified Diet Pattern; Med—Mediterranean; MedDiet—Mediterranean Diet; MF—Muscular Fitness; NA—not applicable; PAI-1—Plasminogen activator inhibitor-1; sICAM-1—Soluble intercellular adhesion molecule-1; sVCAM-1—Soluble Vascular Cell Adhesion Molecule-1; TAC—Total Antioxidant Capacity; TGFβ-1—Transforming growth factor beta 1; TNF-α—Tumour necrosis factor alpha; UDA—Usual dietary advice; VEGF—Vascular Endothelial Growth Factor; WDP—Western Dietary Pattern; *—study examines more than 1 category of dietary intake and appears in both Table 1 and Table 2.

**Table 2:** Summary of studies investigating the associations between food groups and/or nutrients and biological markers of inflammation in children and adolescents.

Author and Country (* also in Table 1)	Sample Characteristics	Pathology (Present or Outcome)	Dietary Intake (Intervention Period If Applicable)	Components Examined	Biological Markers of Inflammation	Main Findings ↑ Increase/Positive Association ↓ Decrease/Negative or Inverse Association ↔ No Association
Experimental—Food Groups and Nutrients						
Hajihashemi et al. (2014) (Iran) [71]	*n* = 44 (22 exp. 22 cont.) 8–15 years 100% female	Overweight/obese Present	Randomized to: Whole-grain diet Control (cross-over trial—6-week cycle, 2 week run-in, 4 week wash-out)	Food group: whole-grains	hs-CRP, sICAM, sVCAM, SAA	Wholegrains v control: hs-CRP, sICAM, SAA ↓ ↔ All other
Jin et al. (2014) (USA) [73]	*n* = 21 Exp. 9, 14.2 ± 0.88, 33% female Cont, 12, 13.0 ± 0.71, 67% female	Non-alcohol fatty liver disease marker (hepatic fat >8%)	Randomized to: Glucose Group Fructose Group (4 weeks)	Food group —sugar sweetened beverages containing 33 g of sugar in either fructose or glucose form	hs-CRP, PAI-1	Post 4 weeks: Glucose: hs-CRP ↓ ↔ All other
Machado et al. (2014) (Brazil) [81]	*n* = 75 Exp 1–25, 52% female Exp 2–25, 48% female Cont. 25, 56% female 13.7 ± 2.1	Overweight Present	Randomized to: Brown Flax Seed (BF) Golden Flax Seed (GF) Control (11 weeks)	Macronutrients: Fibre from provided foods containing brown, golden or no flax seed: coconut cookies, cereal bars, cake, kibbeh, basked stuffed pastries, butter cookies	CRP, interleukin (IL), IL-1B, IL-6, IL-10, INF-y, TNF-α	BF, GF, Cont: TNF-α ↑ All other: ↔
Food Groups—Observational						
Arouca et al. (2017) * (Europe) [47]	*n* = 464 14.79 ± 1.24 53% female	NA	Food Groups Nutrients	Food groups (MedDiet constituents): vegetables, fruits, nuts, pulses, cereals and roots, dairy, fish, meat Macronutrient: MUFA,	CRP, IL-1, IL-2, IL-4, IL-5, IL-6, IL-10, sVCAM-1, sICAM-1, sE-selectin, TNF-α, TGFβ	Dietary Intake: Vegetables and fruit: IL-10 ↑ (all, females), CRP ↑ (males), IL-4 ↓ (all, females), TNF-α ↓ (females) Pulses: IL-5 ↑ (males), IL-6 ↑ (all, males), IL-2 (females) Cereals (refined grains): IL-6, IL-10 ↓ (all) MUFA/SFA: IL-6 (all, females), sVCAM-1 (males), TGFβ-1 (females) ↑ sVCAM-1 (females)↓ Dairy: IL-1 (all, females), IL-5 (all, males), IL-6 (all, males, females), IL-10 (females), TGFβ-1 (females) ↑ Meat: IL-2 (females), IL-10 (all, females) ↑ ↔ All other
Aslam et al. (2020) (Greece) [68]	*n* = 1338 11.5 ± 0.7 51% female	NA	Food Groups	Food group: Dairy products	hs-CRP, IL-6	↔ No associations reported
Cabral et al. (2018) (Portugal) [69]	*n* = 991 13 years 54% female	NA	Food groups	Food groups: vegetables, fruits, starchy, refined grains, whole-grains, dairy, seafood, meat, soft-drink, fast-food, sweets and pastry	hs-CRP	Vegetables/legumes (higher intake): hs-CRP ↓ (obese only)
Gonzales-gil (2015) (Europe) [63]	*n* = 6403 2519 aged 2–6 years 48% female 3884 aged 6–10 years 51% female	NA	Food Groups	Food groups: vegetables, fruits, refined grains, whole-grains, milk, dairy (yoghurt and cheese), fish, meat, egg, drinks, processed foods/snack foods, added sugar—spreads/jam/honey	hs-CRP	Vegetable (increased intake): hs-CRP (males and females) ↓ Refined grains (increased intake): hs-CRP (females) ↑ SSB (increased intake): hs-CRP (females) ↑ ↔ All other
Hagin et al. (2017) (USA) [65]	*n* = 86 12.6 years mean 43% female	Inflammatory Bowel Disease (69% with Crohns Disease) Present	Food Groups	Food groups: vegetables, fruits, snacks (including potato chips, candy, cookies, etc)	CRP	Vegetable intake: CRP ↓ ↔ All other
Han et al. (2015) (Puerto Rico) [67]	*n* = 678 10.5 ± 2.7 47% female	Asthma Present	Food Groups	Food groups: vegetables, fruits, whole-grains, dairy, meat, fats/oils, sweets/soda/snacks	IL-1B, IL-4, IL-6, IL-10, IL-17A, IL-17F, IL-21, IL-22, IL-23, IL-25, IL-31, IL-33, INF-y, TNF-α	Vegetables: IL-17F ↓ Whole-grains: IL-17F ↓ Dairy: IL-17F ↑ Sweets/Soda/Snacks: IL-17F ↑ ↔ All other
Holt et al. (2009) (USA) [66]	*n* = 285 14.9 ± 1.23 46% female	NA	Food Groups Nutrients	Food Groups: vegetables, fruits, fruit juice, french-fried potatoes, legumes Micronutrients: vitamin C, beta-carotene	hs-CRP, IL-6, TNF-α	Total vegetable and fruit: TNF-α, IL-6, ↓ Vegetable: TNF-α ↓ Fruit: hs-CRP ↓ Vitamin C: hs-CRP, IL-6 ↓ Beta-Carotene: IL-6 and TNF-α ↓ ↔ All other
Hur et al. (2012) (USA) [72]	*n* = 4928 15.5 ± 0.1 49% female	NA	Food Groups Nutrients	Food groups: whole-grains	CRP	Whole-grain: CRP ↓ (females)
Kosova et al. (2012) (USA) [74]	*n* = 4880 3–11 years 49% female	NA	Food groups	Food groups: Sugar sweetened beverage intake	CRP	SSB (high intake): CRP ↑ (males)
Navarro (2017) * (Spain) [59]	*n* = 571 6.8 years 53% female	NA	Food Groups Nutrients	Food groups: vegetables, fruits Macronutrients: fat, carbohydrate, protein, fibre Micronutrients: Vitamins: A, E	hs-CRP	High dietary intake: Fruit and Vegetable: hs-CRP ↓ (female) SFA, vitamins A, E: hs-CRP ↑ (female only) Fibre: hs-CRP ↓ (female only) ↔ All other
Qureshi et al. (2009) (USA) [64]	*n* = 4110 11.6 ± 3.3 50% female	NA	Food Groups Nutrients	Food groups: vegetables, fruits, whole-grains, refined grains, dairy, meat/other proteins including eggs Macronutrients: fat, carbohydrate, protein, fibre	hs-CRP	Vegetables, refined grain (low intake): hs-CRP ↑ ↔ All other
Sanjeevi et al. (2018) * (USA) [60]	*n* = 136 12.7 ± 2.6 % female not reported	Type-1 Diabetes Present	Food Groups Nutrients (Secondary analysis of a trial that aimed to increase intake of whole plant foods in experimental group)	Food groups: vegetables, fruits, whole-grains, dairy, meat, eggs, seafood, nuts and seeds, refined grains, sodium, added sugars Macronutrients: fat (SFA)	CRP	↔ No associations reported
Nutrients—Observational						
Aeberli et al. (2006) (Switzerland) [70]	*n* = 79 10.1 ± 2.1 46.83% female	Overweight/obese Present	Nutrients Food Groups	Macronutrients: fats (SFA, PUFA, MUFA, total fat), protein Food Groups: meat intake	hs-CRP, IL-6	Fat (Total fat, % energy as fat): hs-CRP ↑ ↔ All other
Arya et al. (2005) (India) [78]	*n* = 359 18 ± 2.3 13% female	NA	Nutrients	Macronutrients: fat, carbohydrate, protein, fibre	CRP	SFA (high intake): CRP ↑ ↔ All other
Au et al. (2012) (USA) [80]	*n* = 148 10–12 years % female unknown	Cardiometabolic Risk Outcome	Nutrients	Macronutrients: Fat (SFA, MUFA, PUFA), carbohydrate	hs-CRP, IL-6	↔ No associations reported
de Sousa et al. (2017) (Brazil) [89]	*n* = 52 14–19 years 100% male	NA	Nutrients	Micronutrients: Dietary zinc	IL-1B, IL-6, TNF-α	↔ No associations reported
del Mar Bibiloni (2013) * (Spain) [39]	*n* = 219 14.9 ± 1.3 100% female	NA	Nutrients	Macronutrients: fat (SFA, PUFA—linoleic acid, MUFA—oleic acid) Micronutrients: beta-carotene, vitamins A, C, E, manganese, selenium, magnesium	hs-CRP, IL-6, PAI-1, TNF-α,	Dietary intake of: PUFA (linoleic acid): PAI-1 ↓ MUFA (oleic acid), vitamin E: Il-6 ↓ Magnesium: Il-6 ↑ ↔ All other
Harris et al. (2019) (Germany) [77]	*n* = 824 15.2 ± 0.3 53% female	NA	Nutrients	Macronutrients: Fat (SFA)	hs-CRP	SFA: hs-CRP ↓ (males only)
Jaacks et al. (2014) (USA) [82]	*n* = 1405 47.9 ± 43.2 50% female	Type-1 Diabetes Present	Nutrients	Macronutrients: Dietary Fibre	Il-6, CRP	↔ No associations reported
Karampola et al. (2019) * (Greece) [50]	*n* = 142 13.4 ± 1.46 % female not stated	Overweight/obese Present	Nutrients Food Groups	Macronutrients: fat, carbohydrates, protein, fibre Food groups: added sugars	hs-CRP	↔ No associations reported
King et al. (2016) (USA) [88]	*n* = 5007 6–17 years 47% female	NA	Nutrients	Micronutrients: magnesium	CRP	Magnesium (low intake): CRP ↑
Lin et al. (2014) (Europe) [83]	*n* = 1804 14.7 ± 1.2 53% female	NA	Nutrients	Macronutrients: fibre—total fibre, energy-adjusted fibre, water in/soluble fibre	CRP	↔ No associations reported
Miller et al. (2017) (USA) [86]	*n* = 142 15.3 ± 0.1 66.43% female	Overweight/obese Present	Nutrients	Macronutrients: dietary fibre derived from dietary intake information: fat, carbohydrate, added sugars, protein, vegetable, fruit, wholegrains, legumes, refined grains, SSB, snack foods (sweet)	IL-6, IL-8, monocyte chemoattractant protein-1 (MCP1), PAI-1, TNF-α	Total fibre: PAI-1 ↓ Insoluble fibre: PAI-1, MCP-1 ↑ ↔ All other
Oldewage-Theron et al. (2016) (South Africa) [79]	*n* = 232 6–18 years 51.29% female	NA	Nutrients	Macronutrients: fat (PUFA, MUFA, SFA, TFA, linoleic acid) Micronutrients: iron, zinc, magnesium, vitamin C, E	hs-CRP	↔ No associations reported
Parikh et al. (2012) (USA) [85]	*n* = 559 14–18 years 49% female	NA	Nutrients	Macronutrients: fibre	hs-CRP,	Fibre: hs-CRP (both genders) ↓ ↔ All other
Prihaningtyas et al. (2019) (Indonesia) [75]	*n* = 59 13–16 years 46% female	Obesity Present	Nutrients	Macronutrients: fat, carbohydrate, protein	hs-CRP	↔ No associations reported
Swann et al. (2020) (Australia) [84]	*n* = 621 17-years 53%	N/A	Nutrients	Macronutrient: fibre	hs-CRP	↔ No associations reported
Thomas et al. (2007) (UK) [76]	*n* = 164 12–13 years 54% female	NA	Nutrients	Macronutrients: fat (SFA)	hs-CRP	↔ No associations reported
Zhu et al. (2014) (USA) [87]	*n* = 766 14–18 years 50% female	NA	Nutrients	Micronutrients: dietary sodium	hs-CRP, ICAM-1, TNF-α	Sodium intake: TNF-α ↑ ↔ All other

BF—Brown Flax Seed; CRP—C-reactive protein; GF—Golden Flax Seed; hs-CRP—high sensitivity C-reactive protein; IL—Interleukin; IL-1—Interleukin-1; IL-10—Interleukin-10; IL-17A—Interleukin-17A; IL-17F—Interleukin-17F; IL-1b—Interleukin-1b; IL-2—Interleukin-2; IL-21—Interleukin-21; IL-22—Interleukin-22; IL-23—Interleukin-23; IL-25—Interleukin-25; IL-31—Interleukin-31; IL-33—Interleukin-33; IL-4—Interleukin-4; IL-5—Interleukin-5; IL-6—Interleukin-6; IL-B—Interleukin-B; INF-y—Interferon gamma; MCP-1—monocyte chemoattractant protein-1; MUFA—Monounsaturated Fatty Acids; NA—not applicable; PAI-1—Plasminogen activator inhibitor-1; PUFA—Polyunsaturated Fatty Acids; SAA—Serum amyloid-A; sE-selectin—Soluble E-selectin; SFA—Saturated Fatty Acids; sICAM—Soluble intercellular adhesion molecule; sICAM-1—Soluble intercellular adhesion molecule-1; SSB—Sugar Sweetened Beverage; sVCAM—Soluble Vascular Cell Adhesion Molecule; sVCAM-1—Soluble Vascular Cell Adhesion Molecule-1; TFA—Total Fatty Acids; TGFβ-1—Transforming growth factor beta 1; TNF-α—Tumour necrosis factor-alpha; *—study examines more than 1 category of dietary intake and appears in both Table 1 and Table 2.

## Data Availability

Not applicable because it is a systematic review. All data is available in primary studies.

## Supplementary Materials

The following are available online at https://www.mdpi.com/2072-6643/13/2/356/s1. Supplement S1: RoB2.0 and STROBE risk of bias assessment checklists (completed).

## References

[1] Calder P.C., Ahluwalia N., Brouns F., Buetler T., Clement K., Cunningham K., Esposito K., Jö Nsson L.S., Kolb H., Lansink M. (2011). Dietary factors and low-grade inflammation in relation to overweight and obesity commissioned by the ILSI Europe Metabolic Syndrome and Diabetes Task Force. Br. J. Nutr..

[2] Minihane A.M., Vinoy S., McArdle H.J., Kremer B.H.A., Sterkman L., Vafeiadou K., Benedetti M.M., Williams C.M., Calder P.C., Russell W.R. (2015). Low-grade inflammation, diet composition and health: Current research evidence and its translation. Br. J. Nutr..

[3] Giacobbe J., Benoiton B., Zunszain P., Pariante C.M., Borsini A. (2020). The Anti-Inflammatory Role of Omega-3 Polyunsaturated Fatty Acids Metabolites in Pre-Clinical Models of Psychiatric, Neurodegenerative, and Neurological Disorders. Front. Psychiatry.

[4] Kiecolt-Glaser J.K., Derry H.M., Fagundes C.P. (2015). Inflammation: Depression Fans the Flames and Feasts on the Heat. Am. J. Psychiatry.

[5] Zunszain P.A., Hepgul N., Pariante C.M. (2012). Inflammation and Depression. Behavioral Neurobiology of Depression and its Treatment.

[6] Sawyer K.M., Zunszain P.A., Dazzan P., Pariante C.M. (2019). Intergenerational transmission of depression: Clinical observations and molecular mechanisms. Mol. Psychiatry.

[7] Cattaneo A., Ferrari C., Turner L., Mariani N., Enache D., Hastings C., Kose M., Lombardo G., McLaughlin A.P., The Neuroimmunology of Mood Disorders and Alzheimer’s Disease (NIMA) Consortium (2020). Whole-blood expression of inflammasome- and glucocorticoid-related mRNAs correctly separates treatment-resistant depressed patients from drug-free and responsive patients in the BIODEP study. Transl. Psychiatry.

[8] Osimo E.F., Cardinal R.N., Jones P.B., Khandaker G.M. (2018). Prevalence and correlates of low-grade systemic inflammation in adult psychiatric inpatients: An electronic health record-based study. Psychoneuroendocrinology.

[9] Barbaresko J., Koch M., Schulze M.B., Nöthlings U. (2013). Dietary pattern analysis and biomarkers of low-grade inflammation: A systematic literature review. Nutr. Rev..

[10] Furman D., Campisi J., Verdin E., Carrera-Bastos P., Targ S., Franceschi C., Ferrucci L., Gilroy D.W., Fasano A., Miller G.W. (2019). Chronic inflammation in the etiology of disease across the life span. Nat. Med..

[11] Stein D.J., Benjet C., Gureje O., Lund C., Scott K.M., Poznyak V., Van Ommeren M. (2019). Integrating mental health with other non-communicable diseases. BMJ.

[12] Bennett J.M., Reeves G., Billman G.E., Sturmberg J.P. (2018). Inflammation–Nature’s way to efficiently respond to all types of challenges: Implications for understanding and managing “the epidemic” of chronic diseases. Front. Med..

[13] Singh J., Merrill E.D., Sandesara P.B., Schoeneberg L., Dai H., Raghuveer G. (2015). Vitamin D, low-grade inflammation and cardio-vascular risk in young children: A pilot study. Pediatr. Cardiol..

[14] Amaral G.A., Alves J.D., Honorio-França A.C., Fagundes D.L., Araujo G.G., Lobato N.S., Lima V.V., Giachini F.R. (2020). Interleukin 1-beta is Linked to Chronic Low-Grade Inflammation and Cardiovascular Risk Factors in Overweight Adolescents. Endocr. Metab. Immune Disord. Drug Targets.

[15] Al-Hamad D., Raman V. (2017). Metabolic syndrome in children and adolescents. Transl. Pediatr..

[16] Reinehr T. (2019). Inflammatory markers in children and adolescents with type 2 diabetes mellitus. Clin. Chim. Acta.

[17] Stroescu R.F., Mărginean O., Bizerea T., Gafencu M., Voicu A., Doroș G. (2019). Adiponectin, leptin and high sensitivity C-reactive protein values in obese children—Important markers for metabolic syndrome?. J. Pediatr. Endocrinol. Metab..

[18] Marx W., Lane M., Hockey M., Aslam H., Berk M., Walder K., Borsini A., Firth J., Pariante C.M., Berding K. (2020). Diet and depression: Exploring the biological mechanisms of action. Mol. Psychiatry.

[19] Berk M., Williams L.J., Jacka F.N., O’Neil A., Pasco J.A., Moylan S., Allen N.B., Stuart A.L., Hayley A.C., Byrne M.L. (2013). So depression is an inflammatory disease, but where does the inflammation come from?. BMC Med..

[20] Grosso G., Mistretta A., Frigiola A., Gruttadauria S., Biondi A., Basile F., Vitaglione P., D’Orazio N., Galvano F. (2013). Mediterranean Diet and Cardiovascular Risk Factors: A Systematic Review. Crit. Rev. Food Sci. Nutr..

[21] Silveira B.K.S., Oliveira T.M.S., Andrade P.A., Hermsdorff H.H.M., Rosa C.d.O.B., Franceschini S.d.C.C. (2018). Dietary Pattern and Macro-nutrients Profile on the Variation of Inflammatory Biomarkers: Scientific Update. Cardiol. Res. Pract..

[22] Sun C.-H., Li Y., Zhang Y., Zhou X.-L., Wang F. (2011). The effect of vitamin–mineral supplementation on CRP and IL-6: A systemic review and meta-analysis of randomised controlled trials. Nutr. Metab. Cardiovasc. Dis..

[23] Esposito K., Marfella R., Ciotola M., Di Palo C., Giugliano F., Giugliano G., D’Armiento M., D’Andrea F., Giugliano D. (2004). Effect of a Mediterranean-style diet on endothelial dysfunction and markers of vascular inflammation in the metabolic syndrome: A randomized trial. J. Am. Med Assoc..

[24] Giugliano D., Ceriello A., Esposito K. (2006). The effects of diet on inflammation: Emphasis on the metabolic syndrome. J. Am. Coll. Cardiol..

[25] Kohlboeck G., Sausenthaler S., Standl M., Koletzko S., Bauer C.P., Von Berg A., Berdel D., Krämer U., Schaaf B., Lehmann I. (2012). Food Intake, Diet Quality and Behavioral Problems in Children: Results from the GINI-plus/LISA-plus Studies. Ann. Nutr. Metab..

[26] Jacka F.N., Kremer P.J., Berk M., De Silva-Sanigorski A.M., Moodie M., Leslie E.R., Pasco J.A., Swinburn B.A. (2011). A Prospective Study of Diet Quality and Mental Health in Adolescents. PLoS ONE.

[27] Jacka F.N., Kremer P., Leslie E., Berk M., Patton G., Toumbourou J.W., Williams J.W. (2010). Associations Between Diet Quality and Depressed Mood in Adolescents: Results from the Australian Healthy Neighbourhoods Study. Aust. N. Z. J. Psychiatry.

[28] Van der Velde L.A., Nguyen A.N., Schoufour J.D., Geelen A., Jaddoe V.W., Franco O.H., Voortman T. (2019). Diet quality in childhood: The Generation R Study. Eur. J. Nutr..

[29] Lassale C., Batty G.D., Baghdadli A., Jacka F., Sánchez-Villegas A., Kivimäki M., Akbaraly T. (2019). Healthy dietary indices and risk of depressive outcomes: A systematic review and meta-analysis of observational studies. Mol. Psychiatry.

[30] Saugo E., Lasalvia A., Bonetto C., Cristofalo D., Poli S., Bissoli S., Bertani M., Lazzarotto L., Gardellin F., Ceccato E. (2020). Dietary habits and physical activity in first-episode psychosis patients treated in community services. Effect on early anthropometric and cardio-metabolic alterations. Schizophr. Res..

[31] Hilger-Kolb J., Bosle C., Motoc I., Hoffmann K. (2017). Associations between dietary factors and obesity-related biomarkers in healthy children and adolescents—A systematic review. Nutr. J..

[32] Suhett L.G., Hermsdorff H.H.M., Cota B.C., Ribeiro S.A.V., Shivappa N., Hebert J.R., Franceschini S., De Novaes J.F. (2021). Dietary inflammatory potential, cardiometabolic risk and inflammation in children and adolescents: A systematic review. Crit. Rev. Food Sci. Nutr..

[33] Rocha N.P., Milagres L.C., Longo G.Z., Ribeiro A.Q., Novaes J.F.d. (2017). Association between dietary pattern and cardiometabolic risk in children and adolescents: A systematic review. J. Pediatr..

[34] Moher D., Liberati A., Tetzlaff J., Altman D.G. (2010). Preferred reporting items for systematic reviews and meta-analyses: The PRISMA statement. Int. J. Surg..

[35] Von Elm E., Altman D.G., Egger M., Pocock S.J., Gøtzsche P.C., Vandenbroucke J.P. (2007). The Strengthening the Reporting of Observa-tional Studies in Epidemiology (STROBE) statement: Guidelines for reporting observational studies. Ann. Intern Med..

[36] Higgins J.P., Green S. (2011). Cochrane Handbook for Systematic Reviews of Interventions.

[37] Oddy W.H., Allen K.L., Trapp G.S., Ambrosini G.L., Black L.J., Huang R.-C., Rzehak P., Runions K.C., Pan F., Beilin L.J. (2018). Dietary patterns, body mass index and inflammation: Pathways to depression and mental health problems in adolescents. Brain Behav. Immun..

[38] Sen S., Rifas-Shiman S.L., Shivappa N., Wirth M.D., Hebert J.R., Gold D.R., Gillman M.W., Oken E. (2017). Associations of prenatal and early life dietary inflammatory potential with childhood adiposity and cardiometabolic risk in Project Viva. Pediatr. Obes..

[39] Del Mar Bibiloni M., Maffeis C., Llompart I., Pons A., Tur J.A. (2013). Dietary factors associated with subclinical inflammation among girls. Eur. J. Clin. Nutr..

[40] Liese A.D., Ma X., Ma X., Mittleman M.A., Catherine P., Standiford D.A., Lawrence J.M., Pihoker C., Marcovina S.M., Mayer-Davis E.J. (2018). Dietary quality and markers of inflammation: No association in youth with type 1 diabetes. J. Diabetes Its Complicat..

[41] Saneei P., Hashemipour M., Kelishadi R., Esmaillzadeh A. (2014). The Dietary Approaches to Stop Hypertension (DASH) Diet Affects Inflammation in Childhood Metabolic Syndrome: A Randomized Cross-Over Clinical Trial. Ann. Nutr. Metab..

[42] Damsgaard C.T., Papadaki A., Jensen S.M., Ritz C., Dalskov S.-M., Hlavaty P., Saris W., Martínez J.A., Handjieva-Darlenska T., Andersen M.R. (2013). Higher Protein Diets Consumed Ad Libitum Improve Cardiovascular Risk Markers in Children of Overweight Parents from Eight European Countries. J. Nutr..

[43] Iannuzzi A., Licenziati M.R., Vacca M., De Marco D., Cinquegrana G., Laccetti M., Bresciani A., Covetti G., Iannuzzo G., Rubba P. (2009). Comparison of two diets of varying glycemic index on carotid subclinical atherosclerosis in obese children. Hear. Vessel..

[44] Parillo M., Licenziati M.R., Vacca M., De Marco D., Iannuzzi A. (2011). Metabolic changes after a hypocaloric, low-glycemic-index diet in obese children. J. Endocrinol. Investig..

[45] Rouhani M.H., Kelishadi R., Hashemipour M., Esmaillzadeh A., Surkan P.J., Keshavarz A., Azadbakht L. (2016). The Impact of a Low Glycemic Index Diet on Inflammatory Markers and Serum Adiponectin Concentration in Adolescent Overweight and Obese Girls: A Randomized Clinical Trial. Horm. Metab. Res..

[46] Agostinis-Sobrinho C., Ramírez-Vélez R., García-Hermoso A., Rosário R., Moreira C., Lopes L., Martinkenas A., Mota J., Santos R. (2018). The combined association of adherence to Mediterranean diet, muscular and cardiorespiratory fitness on low-grade inflammation in adolescents: A pooled analysis. Eur. J. Nutr..

[47] Arouca A., Michels N., Moreno L.A., González-Gil E.M., Marcos A., Gómez S., Díaz L.E., Widhalm K., Molnár D., Manios Y. (2018). Asso-ciations between a Mediterranean diet pattern and inflammatory biomarkers in European adolescents. Eur. J. Nutr..

[48] De Carvalho K.M.B., Ronca D.B., Michels N., Huybrechts I., Cuenca-García M., Marcos A., Molnar D., Dallongeville J., Manios Y., Schaan B. (2018). Does the Mediterranean Diet Protect against Stress-Induced Inflammatory Activation in European Adolescents? The HELENA Study. Nutrients.

[49] Douros K., Thanopoulou M.-I., Boutopoulou B., Papadopoulou A., Papadimitriou A., Fretzayas A., Priftis K.N. (2019). Adherence to the Mediterranean diet and inflammatory markers in children with asthma. Allergol. Immunopathol..

[50] Karampola M., Argiriou A., Hitoglou-Makedou A. (2019). Study on dietary constituents, hs-CRP serum levels and investigation of correlation between them in excess weight adolescents. Hippokratia.

[51] Lazarou C., Panagiotakos D., Chrysohoou C., Andronikou C., Matalas A.-L. (2010). C-Reactive protein levels are associated with adiposity and a high inflammatory foods index in mountainous Cypriot children. Clin. Nutr..

[52] Sureda A., Bibiloni M.D.M., Julibert A., Bouzas C., Argelich E., Llompart I., Pons A., Tur J.A. (2018). Adherence to the Mediterranean Diet and Inflammatory Markers. Nutrients.

[53] Çağiran Yilmaz F., Çağiran D., Özçelik A.Ö. (2019). Adolescent Obesity and Its Association with Diet Quality and Cardiovascular Risk Factors. Ecol. Food Nutr..

[54] Khayyatzadeh S.S., Bagherniya M., Fazeli M., Khorasanchi Z., Bidokhti M.S., Ahmadinejad M., Khoshmohabbat S., Arabpour M., Afkhamizadeh M., Ferns G.A. (2018). A Western dietary pattern is associated with elevated level of high sensitive C-reactive protein among adolescent girls. Eur. J. Clin. Investig..

[55] Almeida-De-Souza J., Santos R., Barros R., Abreu S., Moreira C., Lopes L., Mota J., Moreira P. (2017). Dietary inflammatory index and inflammatory biomarkers in adolescents from LabMed physical activity study. Eur. J. Clin. Nutr..

[56] Coheley L.M., Shivappa N., Hebert J.R., Lewis R.D. (2019). Dietary inflammatory index® and cortical bone outcomes in healthy ado-lescent children. Osteoporos. Int..

[57] Seremet Kurklu N., Karatas Torun N., Ozen Kucukcetin I., Akyol A. (2020). Is there a relationship between the dietary inflammatory index and metabolic syndrome among adolescents?. J. Pediatr. Endocrinol. Metab. JPEM.

[58] Shivappa N., Hebert J.R., Marcos A., Diaz L.-E., Gomez S., Nova E., Michels N., Arouca A., González-Gil E., Frederic G. (2017). As-sociation between dietary inflammatory index and inflammatory markers in the HELENA study. Mol. Nutr. Food Res..

[59] Navarro P., De Dios O., Jois A., Gavela-Pérez T., Gorgojo L., Martín-Moreno J.M., Soriano-Guillén L., Garcés C. (2017). Vegetable and Fruit Intakes Are Associated with hs-CRP Levels in Pre-Pubertal Girls. Nutrients.

[60] Sanjeevi N., Lipsky L.M., Nansel T.R. (2018). Cardiovascular Biomarkers in Association with Dietary Intake in a Longitudinal Study of Youth with Type 1 Diabetes. Nutrients.

[61] Chan S., Ping-Delfos W.L., Beilin L.J., Oddy W.H., Burrows S., Mori T.A. (2015). Use of the Dietary Guideline Index to assess cardi-ometabolic risk in adolescents. Br. J. Nutr..

[62] Vyncke K.E., Huybrechts I., Dallongeville J., Mouratidou T., Van Winckel M.A., García M.L.C., Ottevaere C., González-Gross M., Moreno L.A., Kafatos A.G. (2013). Intake and serum profile of fatty acids are weakly correlated with global dietary quality in European adolescents. Nutrients.

[63] Gonzalez-Gil E.M., Santabárbara J., Russo P., Ahrens W., Claessens M., Lissner L., Börnhorst C., Krogh V., Iacoviello L., Molnar D. (2015). Food intake and inflammation in European children: The IDEFICS study. Eur. J. Nutr..

[64] Qureshi M.M., Singer M.R., Moore L.L. (2009). A cross-sectional study of food group intake and C-reactive protein among children. Nutr. Metab..

[65] Hagin S., Lobato D.J., Sands B.E., Korzenik J.R., Merrick M., Shah S.A., Bancroft B., Bright R., Law M., Moniz H. (2017). Dietary be-haviors in newly diagnosed youth with inflammatory bowel disease. Child Health Care..

[66] Holt E.M., Steffen L.M., Moran A., Basu S., Steinberger J., Ross J.A., Hong C.-P., Sinaiko A.R. (2009). Fruit and Vegetable Consumption and Its Relation to Markers of Inflammation and Oxidative Stress in Adolescents. J. Am. Diet. Assoc..

[67] Han Y.-Y., Forno E., Brehm J.M., Acostaperez E., Álvarez M., Colón-Semidey A., Rivera-Soto W., Campos H., Litonjua A.A., Alcorn J.F. (2015). Diet, interleukin-17, and childhood asthma in Puerto Ricans. Ann. Allergy Asthma Immunol..

[68] Aslam H., Jacka F.N., Marx W., Karatzi K., Mavrogianni C., Karaglani E., Mohebbi M., Pasco J.A., O’Neil A., Berk M. (2020). The Associations between Dairy Product Consumption and Biomarkers of Inflammation, Adipocytokines, and Oxidative Stress in Children: A Cross-Sectional Study. Nutrients.

[69] Cabral M., Araújo J., Lopes C., Ramos E. (2018). Food intake and high-sensitivity C-reactive protein levels in adolescents. Nutr. Metab. Cardiovasc. Dis..

[70] Aeberli I., Molinari L., Spinas G., Lehmann R., L’Allemand D., Zimmermann M.B. (2006). Dietary intakes of fat and antioxidant vitamins are predictors of subclinical inflammation in overweight Swiss children. Am. J. Clin. Nutr..

[71] Hajihashemi P., Azadbakht L., Hashemipor M., Kelishadi R., Esmaillzadeh A. (2014). Whole-grain intake favorably affects markers of systemic inflammation in obese children: A randomized controlled crossover clinical trial. Mol. Nutr. Food Res..

[72] Hur I.Y., Reicks M. (2012). Relationship between Whole-Grain Intake, Chronic Disease Risk Indicators, and Weight Status among Adolescents in the National Health and Nutrition Examination Survey, 1999–2004. J. Acad. Nutr. Diet..

[73] Jin R., Welsh J.A., Le N.-A., Holzberg J., Sharma P., Martin D.R., Vos M.B. (2014). Dietary fructose reduction improves markers of cardi-ovascular disease risk in Hispanic-American adolescents with NAFLD. Nutrients.

[74] Kosova E.C., Auinger P., Bremer A.A. (2013). The Relationships between Sugar-Sweetened Beverage Intake and Cardiometabolic Markers in Young Children. J. Acad. Nutr. Diet..

[75] Prihaningtyas R., Widjaja N., Irawan R., Hanindita M., Hidajat B. (2019). Dietary Intakes and High Sensivity crp (hsCRP) in Adolescents with Obesity. Carpathian J. Food Sci. Technol..

[76] Thomas N.-E., Baker J.S., Graham M.R., Cooper S.-M., Davies B. (2008). C-reactive protein in schoolchildren and its relation to adiposity, physical activity, aerobic fitness and habitual diet. Br. J. Sports Med..

[77] Harris C., Filipiak-Pittroff B., Berdel D., Bauer C.-P., Schikowski T., Koletzko S., Heinrich J., Schulz H., Standl M. (2019). Dietary saturated fat and low-grade inflammation modified by accelerometer-measured physical activity in adolescence: Results from the GINIplus and LISA birth cohorts. BMC Public Health.

[78] Arya S., Isharwal S., Misra A., Pandey R.M., Rastogi K., Vikram N., Dhingra V., Chatterjee A., Sharma R., Luthra K. (2006). C-reactive protein and dietary nutrients in urban Asian Indian adolescents and young adults. Nutrition.

[79] Oldewage-Theron W., Kruger R. (2016). The association between diet quality and subclinical inflammation among children aged 6–18 years in the Eastern Cape, South Africa. Public Health Nutr..

[80] Au L.E., Economos C.D., Goodman E., Houser R.F., Must A., Chomitz V.R., Morgan E.H., Sacheck J.M. (2012). Dietary Intake and Cardi-ometabolic Risk in Ethnically Diverse Urban Schoolchildren. J. Acad. Nutr. Diet..

[81] Machado A.M., De Paula H., Cardoso L.D., Costa N.M.B. (2015). Effects of brown and golden flaxseed on the lipid profile, glycemia, inflammatory biomarkers, blood pressure and body composition in overweight adolescents. Nutrients.

[82] Jaacks L.M., Crandell J., Liese A.D., Lamichhane A.P., Bell R.A., Dabelea D., D’Agostino R.B., Dolan L.M., Marcovina S., Reynolds K. (2014). No association of dietary fiber intake with inflammation or arterial stiffness in youth with type 1 diabetes. J. Diabetes Its Complicat..

[83] Lin Y., Huybrechts I., Vereecken C., Mouratidou T., Valtueña J., Kersting M., González-Gross M., Bolca S., Wärnberg J., Cuenca-García M. (2014). Dietary fiber intake and its association with indicators of adiposity and serum biomarkers in European adolescents: The HELENA study. Eur. J. Nutr..

[84] Swann O.G., Breslin M., Kilpatrick M., O’Sullivan T.A., Mori T.A., Beilin L.J., Oddy W.H. (2021). Dietary fibre intake and its association with inflammatory markers in adolescents. Br. J. Nutr..

[85] Parikh S., Pollock N.K., Bhagatwala J., Guo D.-H., Gutin B., Zhu H., Dong Y. (2012). Adolescent Fiber Consumption Is Associated with Visceral Fat and Inflammatory Markers. J. Clin. Endocrinol. Metab..

[86] Miller S.J., Batra A.K., Shearrer G.E., House B.T., Cook L.T., Pont S.J., Goran M.I., Davis J.N. (2016). Dietary fibre linked to decreased in-flammation in overweight minority youth. Pediatr. Obes..

[87] Zhu H., Pollock N.K., Kotak I., Gutin B., Wang X., Bhagatwala J., Parikh S., Harshfield G.A., Dong Y. (2014). Dietary Sodium, Adiposity, and Inflammation in Healthy Adolescents. Pediatrics.

[88] King D.E., Mainous A.G., Geesey M.E., Ellis T. (2007). Magnesium intake and serum C-reactive protein levels in children. Magnes Res..

[89] De Sousa A.F., de Andrade Mesquita L.S., Cruz K.J.C., de Oliveira A.R.S., Morais J.B.S., Severo J.S., Beserra J.B., do Nascimento Nogueira N., do Nascimento Marreiro D. (2019). No Relation Between Zinc Status and Inflammatory Biomarkers in Adolescent Judokas. Int. J. Vitam. Nutr. Res..

[90] Zhang R., Chen J., Zheng H., Li Y., Huang H., Liang Z., Jiang H., Sun J. (2019). Effects of medium chain triglycerides on body fat dis-tribution and adipocytokine levels in children with acute lymphoblastic leukemia under chemotherapy. Medicine.

[91] Moeller S.M., Reedy J., Millen A.E., Dixon L.B., Newby P., Tucker K.L., Krebs-Smith S.M., Guenther P.M. (2007). Dietary Patterns: Challenges and Opportunities in Dietary Patterns Research. J. Am. Diet. Assoc..

[92] Casas R., Sacanella E., Estruch R. (2014). The immune protective effect of the Mediterranean diet against chronic low-grade inflam-matory diseases. Endocr. Metab. Immune Disord.-Drug Targets.

[93] Dai J., Miller A.H., Bremner J.D., Goldberg J., Jones L., Shallenberger L., Buckham R., Murrah N.V., Veledar E., Wilson P.W. (2008). Ad-herence to the Mediterranean diet is inversely associated with circulating interleukin-6 among middle-aged men: A twin study. Circulation.

[94] Davis R., Day A.S., Barrett J., VanLint A., Andrews J.M., Costello S.P., Bryant R.V. (2020). Habitual dietary fibre and prebiotic intake is inadequate in patients with inflammatory bowel disease: Findings from a multicentre cross-sectional study. J. Hum. Nutr. Diet..

[95] Chang J.P.-C., Su K.-P., Mondelli V., Satyanarayanan S.K., Yang H.-T., Chiang Y.-J., Chen H.-T., Pariante C.M. (2019). High-dose eicosa-pentaenoic acid (EPA) improves attention and vigilance in children and adolescents with attention deficit hyperactivity disorder (ADHD) and low endogenous EPA levels. Transl. Psychiatry.

[96] Chang J.P.-C., Su K.-P., Mondelli V., Pariante C.M. (2018). Omega-3 polyunsaturated fatty acids in youths with attention deficit hyper-activity disorder: A systematic review and meta-analysis of clinical trials and biological studies. Neuropsychopharmacology.

[97] Borsini A., Alboni S., Horowitz M.A., Tojo L.M., Cannazza G., Su K.-P., Pariante C.M., Zunszain P.A. (2017). Rescue of IL-1β-induced reduction of human neurogenesis by omega-3 fatty acids and antidepressants. Brain Behav. Immun..

[98] Fogarty A., Lewis S.A., McKeever T.M., Britton J.R. (2009). Is higher sodium intake associated with elevated systemic inflammation? A population-based study. Am. J. Clin. Nutr..

[99] Sacks F.M., Svetkey L.P., Vollmer W.M., Appel L.J., Bray G.A., Harsha D., Obarzanek E., Conlin P.R., Miller E.R., Simons-Morton D.G. (2001). Effects on Blood Pressure of Reduced Dietary Sodium and the Dietary Approaches to Stop Hypertension (DASH) Diet. N. Engl. J. Med..

[100] Schultz H., Ying G.-S., Dunaief J.L., Dunaief D.M. (2019). Rising Plasma Beta-Carotene Is Associated With Diminishing C-Reactive Protein in Patients Consuming a Dark Green Leafy Vegetable–Rich, Low Inflammatory Foods Everyday (LIFE) Diet. Am. J. Lifestyle Med..

[101] Li T., Qiu Y., Yang H.S., Li M.Y., Zhuang X.J., Zhang S.H., Feng R., Chen B.L., He Y., Zeng Z.R. (2020). Systematic review and meta-analysis: Association of a pre-illness Western dietary pattern with the risk of developing inflammatory bowel disease. J. Dig. Dis..

[102] Okręglicka K. (2015). Health effects of changes in the structure of dietary macronutrients intake in western societies. Rocz. Państwowego Zakładu Hig..

[103] Zimmermann M.B., Aeberli I. (2008). Dietary determinants of subclinical inflammation, dyslipidemia and components of the metabolic syndrome in overweight children: A review. Int. J. Obes..

[104] Navarro S.L., Kantor E.D., Song X., Milne G.L., Lampe J.W., Kratz M., White E. (2016). Factors Associated with Multiple Biomarkers of Systemic Inflammation. Cancer Epidemiol. Biomark. Prev..

[105] Cunningham-Rundles S., McNeeley D.F., Moon A. (2005). Mechanisms of nutrient modulation of the immune response. J. Allergy Clin. Immunol..

[106] Zolin S.J., Vodovotz Y., Forsythe R.M., Rosengart M.R., Namas R., Brown J.B., Peitzman A.P., Billiar T.R., Sperry J.L. (2015). The Early Evolving Sex Hormone Enviorment is Associated with Significant Outcome and Inflamatory Response Differences Post-injury. J. Trauma Acute Care Surg.

[107] Da Silva C.T.B., de Abreu Costa M., Kapczinski F., de Aguiar B.W., Salum G.A., Manfro G.G. (2017). Inflammation and internalizing dis-orders in adolescents. Prog. Neuropsychopharmacol. Biol. Psychiatry..

[108] Whitacre C.C. (2001). Sex differences in autoimmune disease. Nat. Immunol..

[109] Ortona E., Pierdominici M., Maselli A., Veroni C., Aloisi F., Shoenfeld Y. (2016). Sex-based differences in autoimmune diseases. Ann. Ist. Super. Sanità.

[110] Ağirbaşli M., Agaoglu N.B., Orak N., Caglioz H., Ocek T., Poci N., Salaj A., Maya S. (2009). Sex hormones and metabolic syndrome in children and adolescents. Metabolism.

[111] Reilly S.M., Saltiel A.R. (2017). Adapting to obesity with adipose tissue inflammation. Nat. Rev. Endocrinol..

[112] Monteiro R., Azevedo I. (2010). Chronic Inflammation in Obesity and the Metabolic Syndrome. Mediat. Inflamm..

[113] Livingstone M.B.E., Robson P.J. (2000). Measurement of dietary intake in children. Proc. Nutr. Soc..

[114] Foster E., Adamson A.J. (2014). Challenges involved in measuring intake in early life: Focus on methods. Proc. Nutr. Soc..

[115] Magarey A., Watson J., Golley R.K., Burrows T., Sutherland R., Mcnaughton S.A., Denney-Wilson E., Campbell K., Collins C. (2011). As-sessing dietary intake in children and adolescents: Considerations and recommendations for obesity research. Int. J. Pediatr. Obes..

[116] Cowan S.F., Leeming E.R., Sinclair A., Dordevic A.L., Truby H., Gibson S.J. (2019). Effect of whole foods and dietary patterns on markers of subclinical inflammation in weight-stable overweight and obese adults: A systematic review. Nutr. Rev..

[117] Potischman N. (2003). Biologic and Methodologic Issues for Nutritional Biomarkers. J. Nutr..

[118] Cunha A.L.P.d., Costa A.C.C.d., Vasconcelos Z., Carmo M.d.G.T.D.O., Chaves C.R.M.d.M. (2018). Fatty acid profile in erythrocytes associated with serum cytokines in pediatric cystic fibrosis patients. Rev. Nutr..

[119] Klein-Platat C., Drai J., Oujaa M., Schlienger J.-L., Simon C. (2005). Plasma fatty acid composition is associated with the metabolic syndrome and low-grade inflammation in overweight adolescents. Am. J. Clin. Nutr..

[120] Berger M.E., Smesny S., Kim S.-W., Davey C.G., Rice S., Sarnyai Z., Schlögelhofer M., Schäfer M.R., Berk M., McGorry P.D. (2017). Omega-6 to omega-3 polyunsaturated fatty acid ratio and subsequent mood disorders in young people with at-risk mental states: A 7-year longitudinal study. Transl. Psychiatry.

[121] Dragsted L.O., Gao Q., Scalbert A., Vergères G., Kolehmainen M., Manach C., Brennan L., Afman L.A., Wishart D.S., Andres-Lacueva C. (2018). Validation of biomarkers of food intake—Critical assessment of candidate biomarkers. Genes Nutr..

[122] Tworoger S.S., Hankinson S.E. (2006). Use of biomarkers in epidemiologic studies: Minimizing the influence of measurement error in the study design and analysis. Cancer Causes Control..

